# Protective role of silibinin against myocardial ischemia/reperfusion injury-induced cardiac dysfunction

**DOI:** 10.7150/ijbs.39259

**Published:** 2020-04-27

**Authors:** Yi-He Chen, Hui Lin, Qian Wang, Jian-Wen Hou, Zhi-Jie Mao, Yi-Gang Li

**Affiliations:** 1 Department of Cardiology, The First Affiliated Hospital of Wenzhou Medical University, 325000, Nanbaixiang, Wenzhou, Zhejiang, China.; 2 Department of Respiratory, The Second Affiliated Hospital and Yuying Children's Hospital of Wenzhou Medical University, 325000, Wenzhou, Zhejiang, China.; 3 Department of Cardiology, Affiliated Xinhua Hospital, Shanghai Jiaotong University (SJTU) School of Medicine, Shanghai, China.

**Keywords:** Myocardial I/R injury, Silibinin, Apoptosis, Oxidative stress, Inflammation, NF-κB

## Abstract

Silibinin is a traditional medicine and utilized for liver protection with antioxidant, anti-inflammation and anti-apoptosis properties. However, its role in myocardial I/R injury and the mechanism involved is currently unknown. In the present study, Silibinin treatment improves cardiac function and limits infarct size, and subsequently inhibits fibrotic remodeling in mice with myocardial I/R injury. Mechanistically, silibinin reduces cardiomyocytes apoptosis, attenuates mitochondrial impairment and endoplasmic reticulum (ER) stress, alleviates ROS generation, neutrophil infiltration and cytokines release. Consistently, silibinin prevents H9C2 cells from hypoxia/reperfusion-induced cell death, oxidative stress and inflammation *in vitro*. Furthermore, H9C2 cells treated with silibinin blocks NF-κB signaling activation by inhibiting IKKα phosphorylation, IκBα degradation and p65 NF-κB nuclear translocation during hypoxia/ reperfusion. In addition, silibinin plus BAY 11-7082 (a selected NF-κB inhibitor) do not provide incremental benefits in improving myocytes apoptosis, oxidative stress and inflammation in comparison with NF-κB signaling inhibition only. Thus, silibinin-mediated cardioprotection in myocardial I/R injury is associated with decreased apoptosis, oxidative stress and inflammatory response through deactivation of NF-κB pathway.

## Introduction

Myocardial infarction is the leading cause of morbidity and mortality worldwide 1. Although early reperfusion with thrombolytic therapy or PCI prevents the myocardium from sustained ischemia. However, restored coronary flow per se paradoxically impairs the cardiac function and leads to detrimental outcomes, which is referred to as myocardial I/R injury 2,3. While the underlying molecular mechanisms of I/R injury remain unknown, emerging evidence highlights the critical role of excessive oxidative stress, severe inflammation and amplified cascade of apoptosis pathway in exacerbating cardiomyocytes loss and myocardial dysfunction 2-4. Therefore, effective treatment targeting the aforementioned pathological process is of pressing need for limiting myocardial damage after I/R injury.

Silibinin, a polyphenolic flavonoid, is the main active component extracted from the seed of silybum marianum (*milk thistle*) or artichoke (*cynara scolymus*) 5. Previous studies have identified the antineoplastic effect of silibinin by its ability to interfere with the cell cycle progression, suppress angiogenesis, induce apoptosis and inhibit metastasis 6-8. While the protective effects of silibinin have also been extensively investigated in the setting of liver injury caused by toxic compounds 9,10. The antihepatotoxic mechanisms may be attributed to the membrane stabilizing, anti-inflammatory, antioxidant, along with anti-apoptosis properties 11. Moreover, silibinin presents neuroprotective activity, cardioprotective activity, anti-fibrotic remodeling and regulates lipid metabolism 12,13. Recently, data from animal studies have demonstrated that silibinin confers protective advantage in improving both liver and cerebral function after I/R, which raises concern about the role of silibinin against reperfusion injury in other tissues, especially in myocardium 14-16. Despite Rao et al 16 have reported that long-term administration of silymarin attenuates the I/R-induced myocardial damage, evidenced by reduced neutrophil infiltration, decreased MDA generation and limited infarct size, as a compound, it is hard to attribute the beneficial effects of silymarin to one of the components. As a major constituent of the silymarin mixture, however, little is known concerning the therapeutic role of silibinin in myocardial I/R injury.

In this study, we hypothesis that silibinin treatment could attenuate the I/R-induced cardiac dysfunction and adverse remodeling. We intend to further clarify the molecular and cellular mechanisms underlying the cardioprotective effects of silibinin in the pathogenesis of myocardial I/R injury.

## Methods

### Animals

C57BL/6 mice (8-12 weeks old, male) were purchased from Wenzhou Medical University and housed under a controlled temperature (25°C) with a 12 h light/dark cycle. All experiments were conducted according to the guidance for the care and use of experimental animals published by NIH (the 8th Edition, NRC 2011) and approved by the Animal Care and Use Committee of Wenzhou Medical University.

### Myocardial I/R model and treatment

The mice model of Myocardial I/R was performed as described previously 17. Male C57BL/6 mice (8-12 weeks old) were anesthetized by 2% isoflurane inhalation and ventilated mechanically. After left thoracotomy, the LAD was reversibly ligated with a slipknot (6-0 silk sutures). After 30 minutes of ischemia, reperfusion was achieved by releasing the slipknot for 24 hours. Sham-operated mice subjected to equivalent procedure with the absence of myocardial ischemia. Mice were randomized to four groups: sham+vehicle group, I/R+vehicle group, sham+silibinin group and I/R+silibinin group. Silibinin (Sigma, powder, S0417) (100mg/kg, dissolved in DMSO) or vehicle was consecutively administered by intraperitoneal injection for 7 days before I/R. To determine anti-fibrotic remodeling of silibinin, mice were consecutively treated by intraperitoneal injection for 7 days before I/R, and continued for 2 weeks post reperfusion injury.

### Measurement of infarct size

To determine the infarct size, LAD was re-occluded 24 hours after reperfusion, 2% Evans blue dye was perfused via the jugular venous. Then the heart was excised, sliced and incubated with 1% TTC. AAR was defined as the area lacking Evans blue staining, and the infarct area was regarded as the white area within the AAR. AAR was quantified as a percentage of the LV (AAR/LV), and Infarct area was calculated as a percentage of the AAR (infarct area/AAR) using Image-Pro Plus software.

### Echocardiographic and hemodynamic analysis

In the model of myocardial I/R, echocardiography had been reported as a more accurate measure of systolic dysfunction 48, thus the mice were anesthetized with 2% isoflurane inhalation, an echocardiographic imaging system (Vevo 770, Visual Sonic, Canada) with 15 MHz transducer was utilized to assess the cardiac function at 24 hours and 2 weeks after reperfusion. Mice were placed in the supine position on a 37°C platform. M-mode images were recorded from the long axis of the left ventricle, then LVEF and FS were measured. Furthermore, hemodynamic analysis was also performed as a complementary assay for reflecting the myocardial contractility 46. Briefly, 24 hours post I/R, the mice were anesthetized, a pressure-monitored catheter (SPR838, Millar Instruments, USA) was inserted into LV through right carotid artery and LV maximum (+dP/dt_max_) and minimum (-dP/dT_min_) were recorded.

### Histology and immunohistochemistry

Hearts were excised, weighted, fixed in 4% paraformaldehyde and embedded in paraffin. 5-μm sections were collected and subjected to sirius red staining to assess collagen deposition. Immunohistochemistry was performed using anti-collagen-Ia1 (Abcam, 1:200), anti-collagen-IIIa1 (Abcam, 1:200), anti-MPO (Abcam, 1:100) antibodies. Then the sections were incubated with biotinylated goat anti-rabbit secondary antibody, detected by DAB.

### Western Blotting

Proteins from heart tissues or cells were extracted as previously described 18. 30 μg of protein was separated by SDS-PAGE gel, transferred to PVDF membranes. After blocking with 5% non-fat milk, the membranes were incubated with primary antibodies against cytochrome c (Cell Signaling, 1:1000), CHOP (Abcam, 1:1000), catalase (Cell Signaling, 1:1000), α-SMA (Abcam, 1:1000), IκBα (Abcam, 1:1000), NF-κB (Cell Signaling, 1:1000), p-NF-κB (Abcam, 1:500), p65 NF-κB (Abcam, 1:1000), lamin A/C (Abcam, 1:500), p-IKKα (Abcam, 1:500), IKKα (Abcam, 1:1000) at 4°C overnight, and followed by incubation with HRP-conjugated secondary antibodies. Immunoreactive bands were visualized with ECL chemiluminescence and quantified by using Image-Pro Plus software.

### Real-time PCR

Total RNA from heart tissues or cells was isolated with TRIzol reagent, reverse transcribed into cDNA using PrimeScript RT kit (Takara, Japan). Real-time PCR was conducted using ABI 7500 Fast (Applied Biosystems, USA) with the primers listed in Table 1. The transcript levels were normalized to GAPDH.

### TUNEL staining

Heart tissues or cell sections were stained using *in situ* TUNEL kit (Roche) according to the manufacturer's instructions. Briefly, sections were treated with 0.1% Triton X-100, followed by incubation of TUNEL, then co-stained with α-actinin and DAPI. Fluorescence images were obtained with Leica DMI3000B microscope and analyzed by using Image-Pro Plus software.

### Measurement of caspase activities

Caspase-3, caspase-9, and caspase-12 activity was measured using respective caspase assay kits (Beyotime Biotechnology, China) 47. Briefly, heart tissues were homogenized, centrifuged to obtain supernatants. Supernatants (100 µg protein) were loaded in 96-well plate, incubated with Ac-DEVD-pNA for 60 min at 37°C, and then quantified by microplate reader according to the manufacturer's instruction.

### Measurement of cTn-I release

24 hours after I/R injury, blood was collected, centrifuged and separated. Serum was used to measure cTn-I using mouse-specific ELISA kit.

### Measurement of ROS generation

ROS production in myocardium after I/R injury was detected as described previously 17. Hearts were excised and placed into OCT. Unfixed cryosections (10μm) were then incubated with DHE for 30 min at 37°C. The fluorescence intensity was measured by a Leica DMI3000B microscope. To determine the ROS production in H9C2 cells subjected to hypoxia/reperfusion, cells were incubated with DCFH-DA (10μm) for 30 minutes at 37°C. Then images were obtained with fluorescent microscope.

### Measurement of MPO activity

MPO activity was measured using a commercial assay kit (Abcam, USA). Heart tissues were homogenized, centrifuged and supernatants was collected. Samples were added into a 96-well plate, incubated with reaction mix and measured at Ex/Em=484/525 nm by microplate reader 49.

### Cell culture and hypoxia/reperfusion model

Rat cardiomyocyte-derived H9C2 cells were cultured (95% O_2_ and 5% CO_2_, 37°C) in DMEM medium containing 10% FBS, 100 U/ml penicillin/streptomycin. For *in vitro* hypoxia/reperfusion experiment, H9C2 cells were incubated in DMEM without glucose under hypoxic condition (1% O_2_) for 6 hours, and then the medium was replaced by normal DMEM and reoxygenated under normoxic condition (95% O_2_) for 12 hours.

Silibinin (50µmol/L), BAY 11-7082 (an NF-κB inhibitor) (20µmol/L) or both was added into the medium 24 hours prior to hypoxia/reperfusion insult.

### Extraction of nuclear protein

Nuclear protein was prepared as described previously 19. Briefly, H9C2 cells were washed, centrifuged and suspended in cytosolic extraction buffer (Beyotime Biotechnology, China). Then the pellets were resuspended in nuclear extract buffer. Resultant supernatants were lysed in RIPA buffer and collected as nuclear protein.

### Measurement of LDH and cell viability

LDH activity was determined using the LDH activity assay kit (Beyotime Biotechnology, China). Briefly, H9C2 cells were cultured in 96-well plate and then exposed to hypoxia/reperfusion. After silibinin treatment for 24 h in DMEM, the cultured medium from H9C2 cells was collected and centrifuged. Supernatants were separated, transferred into another 96-well plate. LDH activity was measured according to the manufacturer's instruction. Cell viability was determined using CCK-8 kit as previously described 18.

### Measurement of cytokines

Supernatants from H9C2 cells and plasma from reperfused mice were extracted and transferred into another 96-well plate. The cytokines were determined using ELISA kit specific for IL-6 and TNF-α according to the manufacturer's instruction (Beyotime Biotechnology, China).

### Statistical analysis

Data were expressed as mean ± SEM. One-way ANOVA with the Tukey post hoc analysis or Student t test was performed for comparisons using Statistical package SPSS version 20.0 (SPSS Inc., IL, USA). A value of *P*<0.05 was considered to be statistically significant.

## Results

### Silibinin improves cardiac function after I/R injury

**Figure 1:**
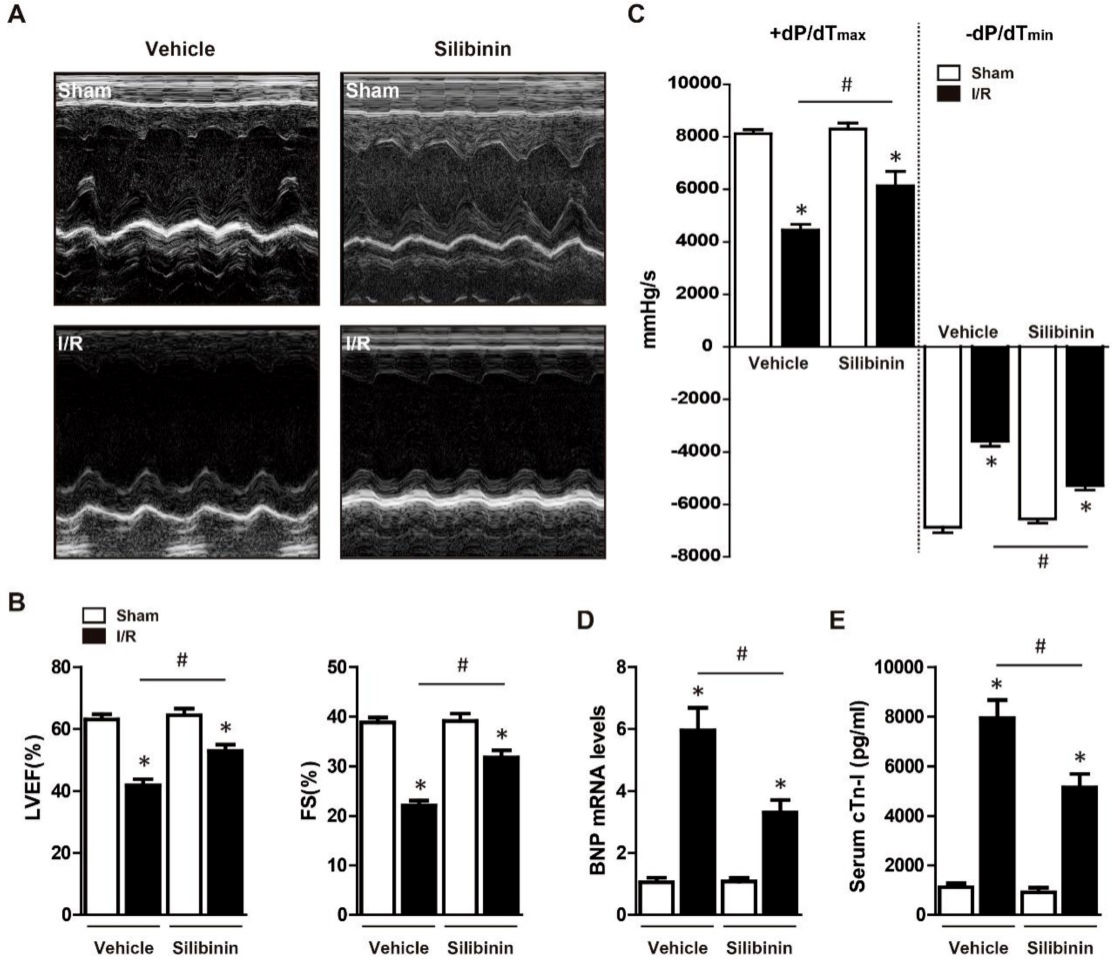
** Effect of silibinin on I/R-induced cardiac dysfunction.** A, Representative images of M-mode echocardiograms obtained at baseline and 24 hours after I/R. B, Quantitative analysis of LVEF, FS through echocardiography (n=6 for each). C, Hemodynamic analysis of +dP/dT_max_ and -dP/dT_min_ (n=6 for each). D, BNP transcription levels determined by real-time PCR (n=6 for each). E, Measurement of serum cTn-I level at 24 hours after I/R (n=6 for each).*p<0.05 vs. Sham+vehicle. ^#^p<0.05 vs. I/R+vehicle.

To determine the protective effects of silibinin on cardiac function, mice were treated with vehicle or silibinin for 7 days prior myocardial I/R injury. Echocardiography was performed after 30 minutes ischemia and 24 hours reperfusion, as shown in Fig. 1A, I/R severely suppressed the LVEF and FS. However, compared with vehicle, silibinin treatment significantly improved I/R-induced cardiac dysfunction (LVEF: 52.9±2.1% vs. 41.9±2.0%; FS: 31.7±1.5% vs. 22.1±1.0%) (Fig. 1B). Subsequently, hemodynamic analysis was conducted to further assess the LV performance, silibinin markedly restored the compromised contractility after I/R injury (+dP/dT_max_: 6131.3±553.4% vs. 4442.8±217.9%; -dP/dT_min_: -5277.3±182.9% vs. -3588.3±193.4%) (Fig. 1C). In line with this, silibinin also inhibited the I/R-induced the expression of BNP (Fig. 1D). Serum cTn-I level, an indicator of myocardial injury, was significantly reduced in mice treated with silibinin (Fig. 1E).

### Silibinin attenuates I/R injury-induced myocardial apoptosis

**Figure 2:**
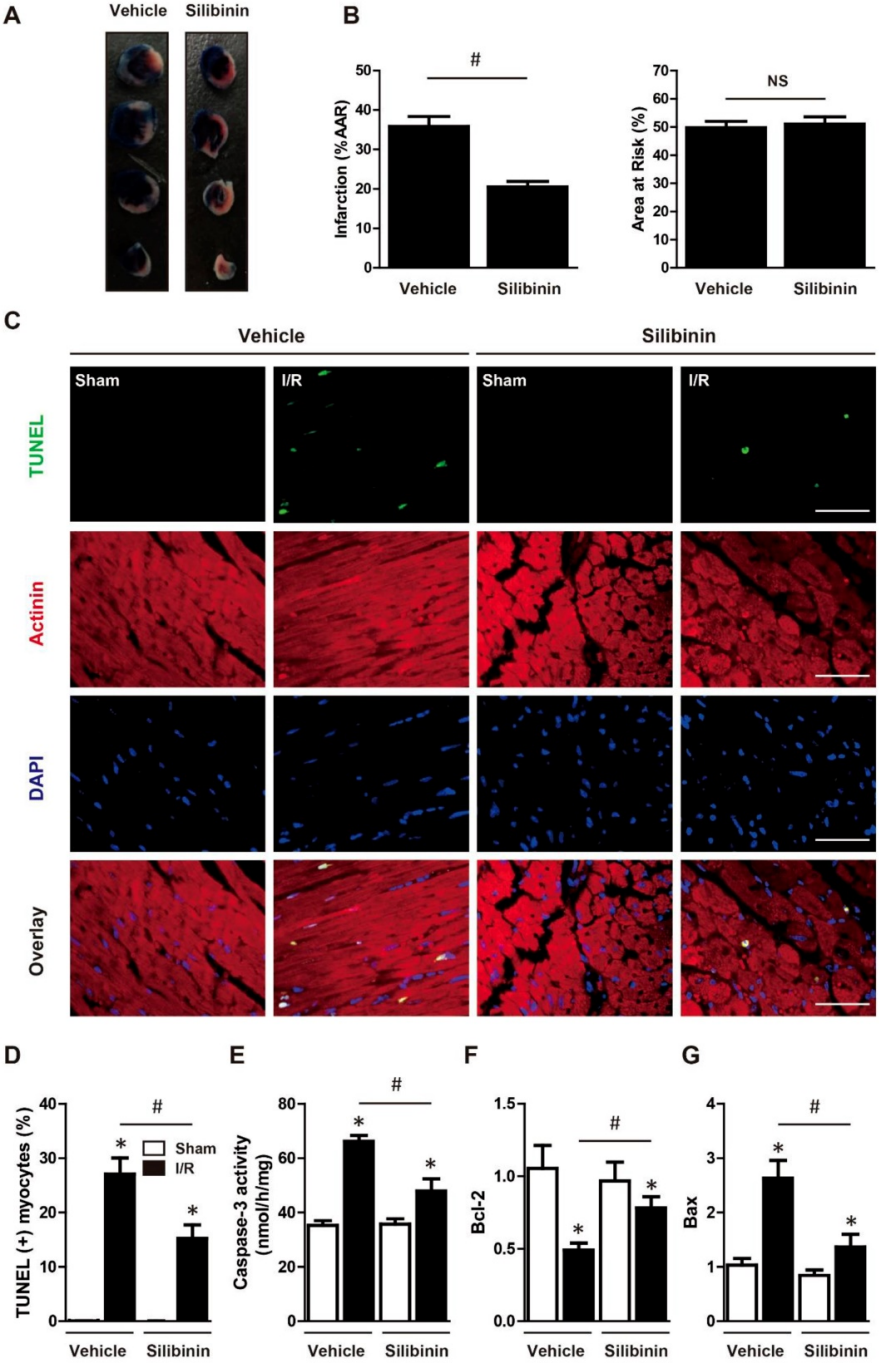
** Silibinin treatment limits infarct size, reduced cardiomyocytes apoptosis after I/R injury.** A, Representative images of transverse heart sections after Evans Blue and TTC double staining. B, Quantitative analysis of infarct area and AAR at 24 hours after I/R (n=6 for each). C, Representative immunofluorescences of TUNEL (green), α-actinin (red), and DAPI (blue) staining in the infarct border zone. D, Quantitative analysis of TUNEL-positive cardiomyocytes in the infarct border zone at 24 hours after I/R (n=6 for each). E, Measurement of caspase-3 activity in reperfused myocardium at 24 hours after I/R (n=6 for each). F and G, Bcl-2 and Bax transcription levels determined by real-time PCR (n=6 for each). Bar=50 μm. *p<0.05 vs. Sham+vehicle. ^#^p<0.05 vs. I/R+vehicle. NS, not significant.

**Figure 3:**
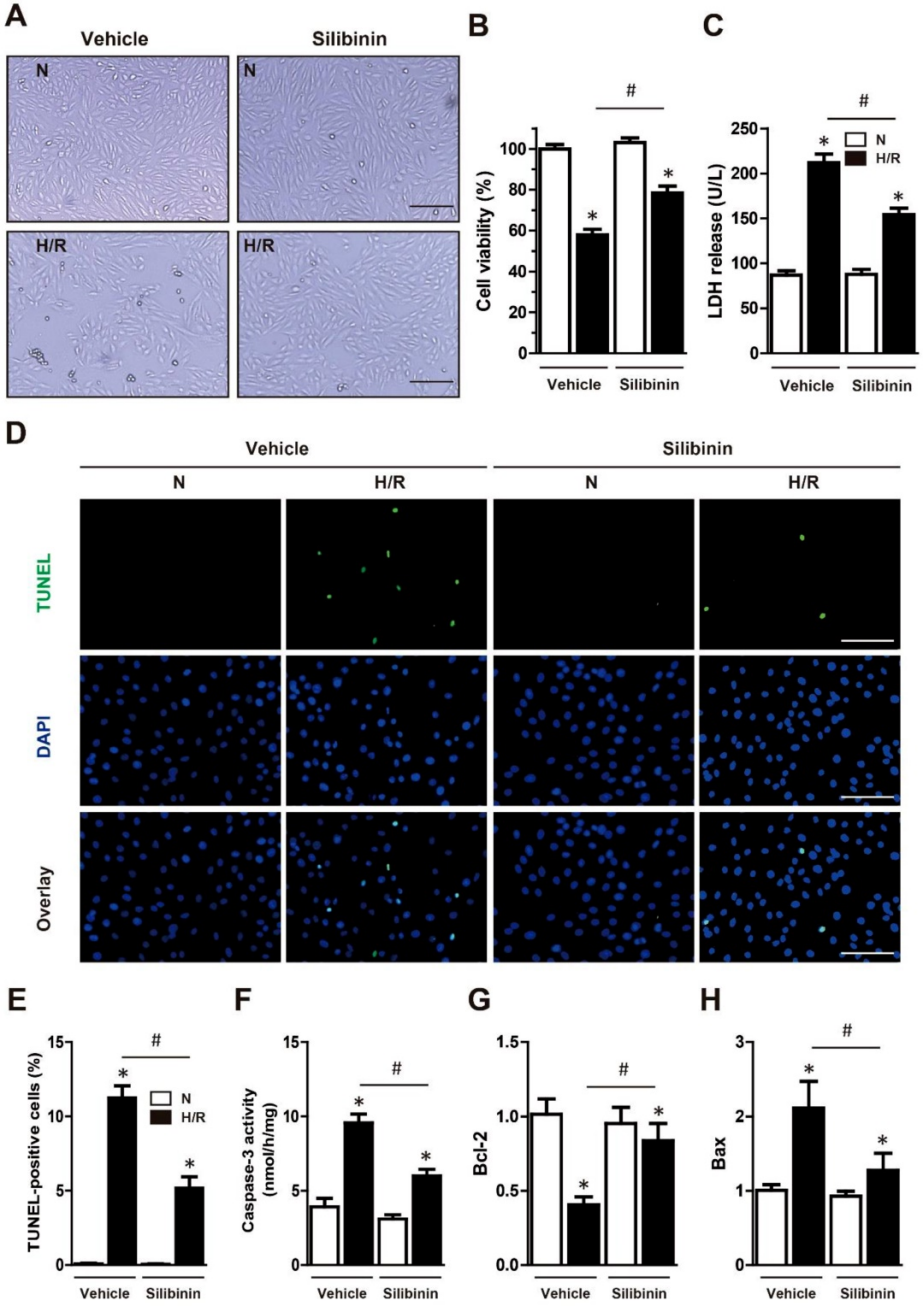
** Silibinin treatment reduces H9C2 cells death subjected to H/R.** A, Representative histological images of H9C2 cells morphology in H/R. Bar=500 μm. B, Cell viability determined by CCK-8 (n=4 for each). C, Measurement of LDH release in H9C2 cells exposed to H/R (n=4 for each). D, Representative immunofluorescences of TUNEL (green), and DAPI (blue) staining in H9C2 cells (n=4 for each). Bar=50 μm. E, Quantitative analysis of TUNEL-positive H9C2 cells in H/R. F, Measurement of caspase-3 activity in H9C2 cells after H/R. G and H, Bcl-2 and Bax transcription levels determined by real-time PCR (n=4 for each). *p<0.05 vs. Sham+vehicle. ^#^p<0.05 vs. H/R+vehicle. H/R, hypoxia/reperfusion.

To determine the susceptibility to I/R injury, infarct size was measured. The AAR was similar in both groups, indicating the same ischemic area and reproducibility of ligature. Consistent with cTn-I level, silibinin treatment significantly decreased infarct size in comparison with vehicle (20.5±1.3% vs. 35.9±2.5%) (Fig. 2A, B). Myocardial apoptosis had been demonstrated to be a key factor underlying I/R injury. Twenty-four hours after injury, heart tissues presented an increase of TUNEL-positive cardiomyocytes in peri-infarct zone, whereas treatment with silibinin remarkably inhibited I/R-induced apoptosis (Fig. 2C, D). Similarly, caspase-3 activity was concomitantly increased after I/R injury and decreased by silibinin (Fig. 2E). Additionally, silibinin treatment impeded the down-regulation of Bcl-2 and up-regulation of Bax following I/R injury (Fig. 2F, G). To further explore the anti-apoptotic effect of silibinin on cardiomyocytes, H9C2 cells were subjected to 6 hours hypoxia and reoxygenated for 12 hours. Silibinin treatment restored the viability of H9C2 cells in hypoxia/reperfusion, as evidenced by visual inspection and increased CCK-8 value (Fig. 3A, B). In parallel, hypoxia/reperfusion-induced apoptosis of H9C2 cells, release of LDH in the medium and activation of caspase-3, however, silibinin treatment *in vitro* reduced the number of TUNEL-positive H9C2 cells, lowered the level of LDH and caspase-3 activity (Fig. 3C-F). Moreover, in accordant with the results from *in vivo* study, dysregulated expression of Bcl-2 and Bax were restored by silibinin in H9C2 cells under hypoxia/reperfusion (Fig. 3G, H).

### Silibinin inhibits I/R injury-induced fibrotic remodeling

**Figure 4:**
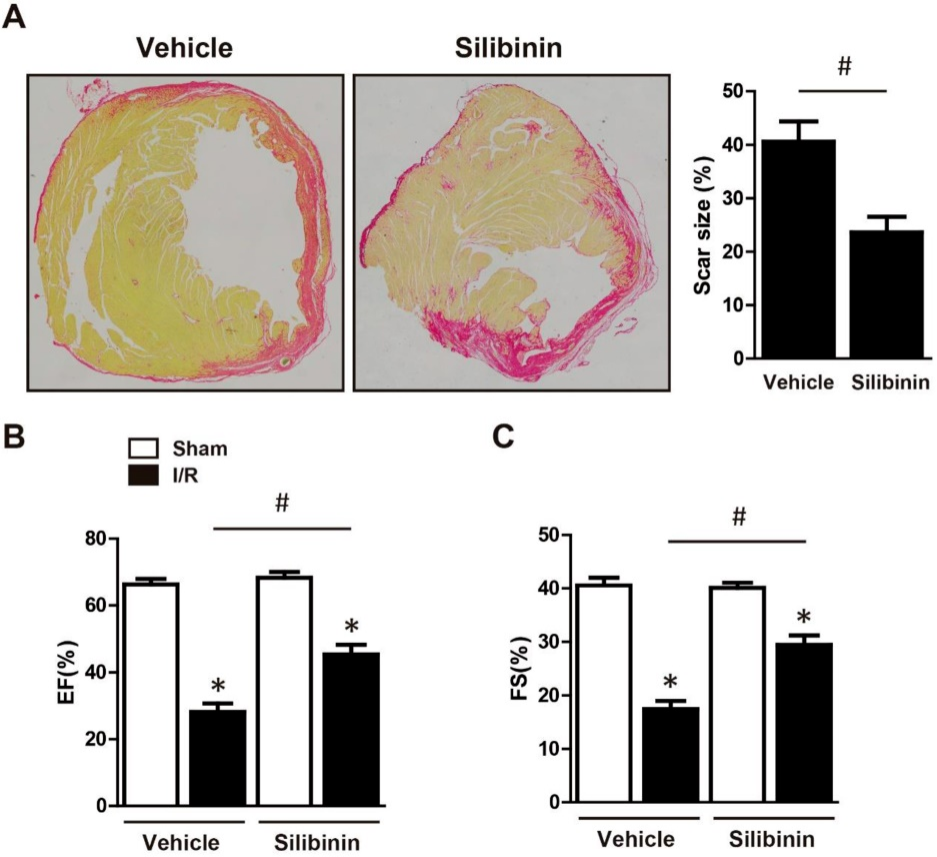
** Silibinin treatment reduced the scar size, improved the cardiac function at 2 weeks post I/R.** A, Representative histological images of the Sirius Red staining and quantitative analysis of scar size (n=6 for each). B, Quantitative analysis of LVEF, FS through echocardiography (n=6 for each). *p<0.05 vs. Sham+vehicle. ^#^p<0.05 vs. I/R+vehicle.

**Figure 5:**
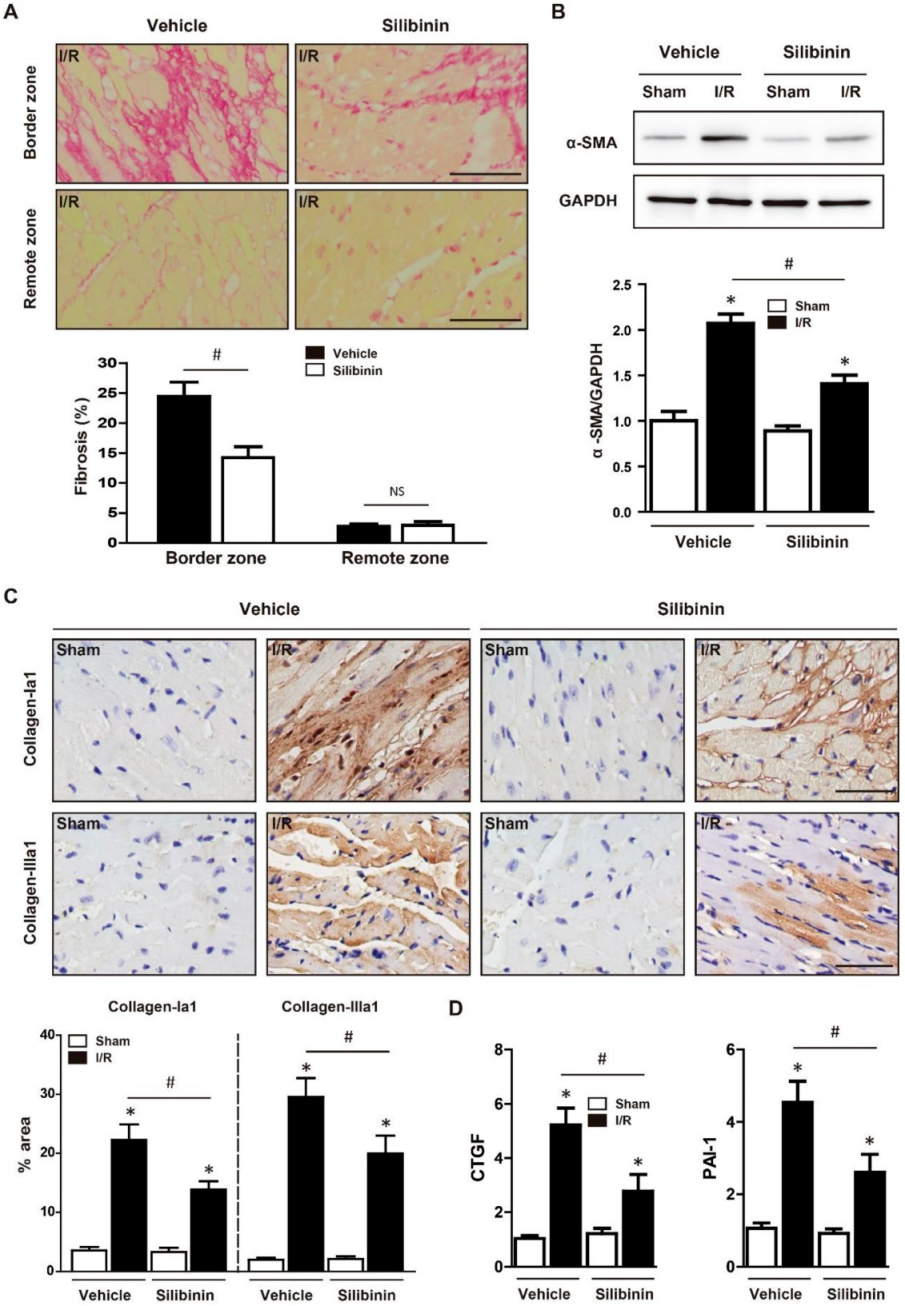
** Silibinin treatment ameliorates fibrotic remodeling after myocardial I/R injury.** A, Representative histological images and quantitative analysis of the Sirius Red staining in the infarct border zone and remote zone at 2 weeks after I/R (n=6 for each). B, Representative WB and quantitative analysis of α-SMA in reperfused myocardium (n=6 for each). C, Representative immunohistochemistry images and quantitative analysis of collagen-Ia1 and collagen-IIIa1 staining in the infarct border zone. D, CTGF and PAI-1 transcription levels determined by real-time PCR (n=6 for each). Bar=50 μm. *p<0.05 vs. Sham+vehicle. ^#^p<0.05 vs. I/R+vehicle. NS, not significant.

Cardiomyocytes apoptosis led to fibroblasts activation, replacement and collagen deposition which eventually aggravated cardiac dysfunction 20. To investigate the effect of silibinin on fibrotic remodeling after I/R injury, mice were consecutively treated with silibinin for 2 weeks post injury and sirius red staining was performed. Notably, Silibinin treatment significantly decreased the scar size (23.6±2.9% vs. 40.6±3.8%) (Fig. 4A) and cardiac fibrosis in the infarct border zone (14.3±1.8% vs. 24.5±5.9%) but not in the remote zone (3.0±1.5% vs. 2.8±0.9%) (Fig. 5A). In accordant with this, I/R-induced expression of α-SMA was also attenuated in silibinin group (Fig. 5B). Collagen-Ia1 and -IIIa1 expression detected by immunohistochemistry was dramatically increased in response to injury, but treatment with silibinin alleviated the collagen deposition (Fig. 5C). Additionally, silibinin significantly reduced the mRNA levels of CTGF and PAI-1 after I/R injury (Fig. 5D). Furthermore, silibinin also presented restored cardiac function 2 weeks after I/R (LVEF: 45.4±2.8% vs. 28.2±2.5%; FS: 29.5±1.7% vs. 17.4±1.5%) (Fig. 4B, C).

### Silibinin inhibits I/R injury-induced mitochondrial dysfunction and ER stress

**Figure 6:**
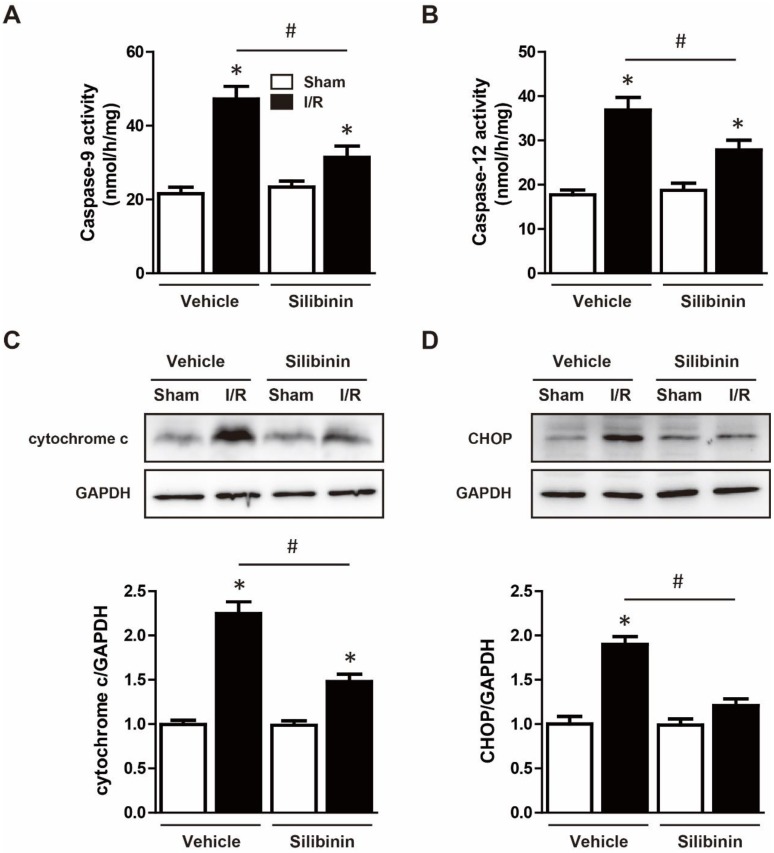
** Silibinin treatment attenuates mitochondrial dysfunction and ER stress.** A and B, Measurement of caspase-9 and caspase-12 activity in reperfused myocardium at 24 hours after I/R (n=6 for each). C and D, Representative WB and quantitative analysis of cytochrome c and CHOP in reperfused myocardium (n=6 for each). *p<0.05 vs. Sham+vehicle. ^#^p<0.05 vs. I/R+vehicle.

Mitochondrial dysfunction and ER stress was the main pathway of apoptosis and thus contributed to I/R injury 21. As expected, I/R activated caspase-9 and caspase-12, administration of silibinin significantly hindered the activation of both caspases (Fig. 6A, B). Furthermore, increased expression of cytochrome c and CHOP also demonstrated the dysfunction of mitochondria and ER, while silibinin treatment attenuated the expression of cytochrome c and CHOP when compared with vehicle (Fig. 6C, D).

### Silibinin reduces I/R injury-induced oxidative stress

**Figure 7:**
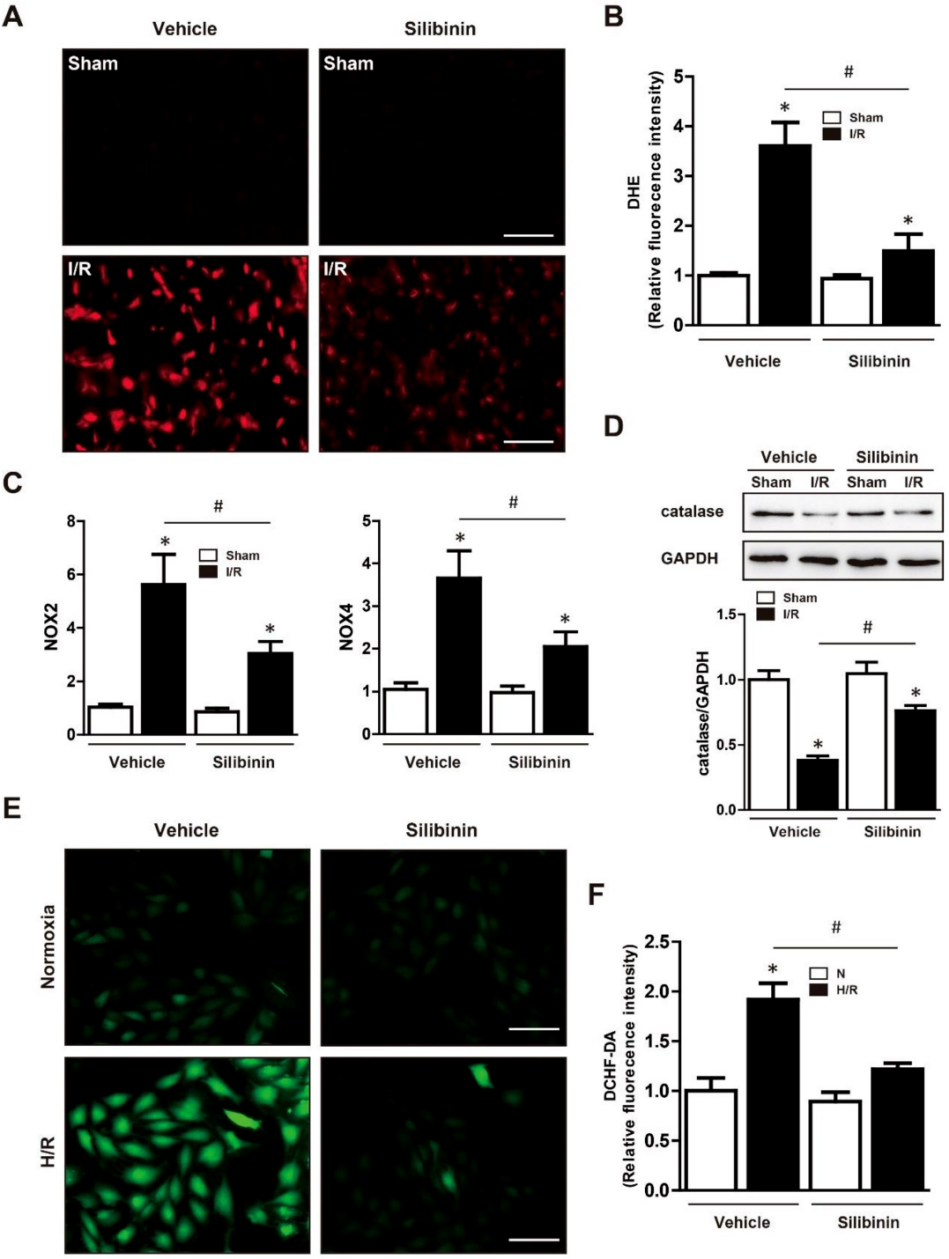
** Silibinin treatment alleviates I/R-induced oxidative stress.** A, Representative images of the DHE staining in the infarct border zone. B, Fluorescence intensity of DHE measured in the infarct border zone at 24 hours after I/R (n=6 for each). C, NOX2 and NOX4 transcription levels determined by real-time PCR (n=6 for each). D, Representative WB and quantitative analysis of catalase in reperfused myocardium (n=6 for each). Bar=50 μm. *p< 0.05 vs. Sham+vehicle. ^#^p< 0.05 vs. I/R+vehicle. E, Representative images of DCHF-DA staining in H9C2 cells. F, Fluorescence intensity of DCHF-DA measured in H9C2 cells exposed to H/R (n=4 for each). Bar=50 μm. *p<0.05 vs. Sham+vehicle. ^#^p<0.05 vs. H/R+vehicle. H/R, hypoxia/reperfusion.

**Figure 8:**
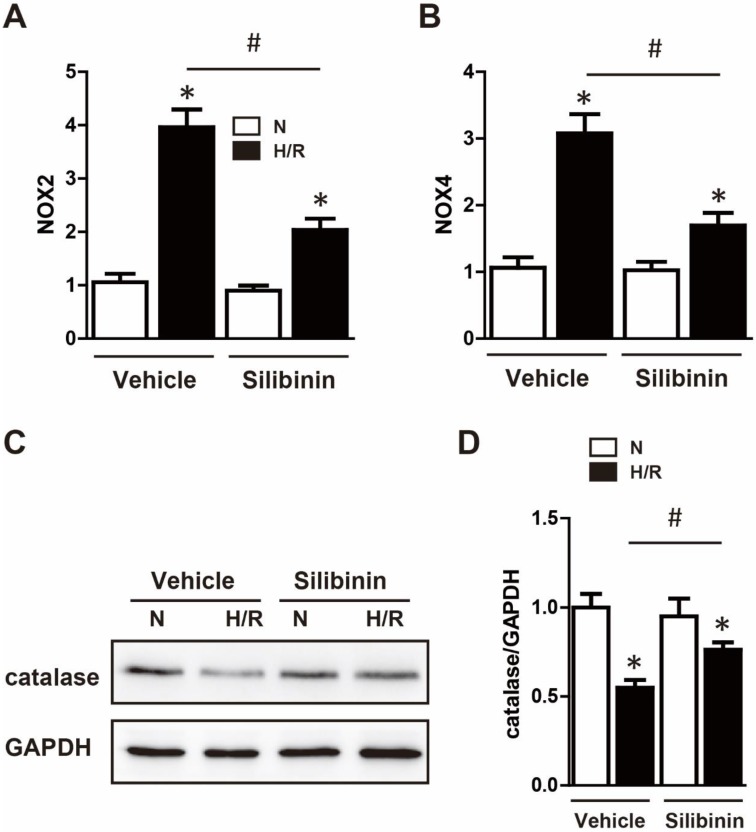
** Silibinin treatment diminished the gene expression of NOX2 and NOX4, blunted the reduction of catalase in H9C2 cells under H/R.** A and B, NOX2 and NOX4 transcription levels determined by real-time PCR in H9C2 cells exposed to H/R (n=4 for each). C and D, Representative WB and quantitative analysis of catalase (n=4 for each). *p<0.05 vs. Sham+vehicle. *p<0.05 vs. Sham+vehicle. ^#^p<0.05 vs. H/R+vehicle. NS, not significant. H/R, hypoxia/reperfusion.

Mitochondrial dysfunction resulted in a surge of ROS release, which caused oxidative stress and deteriorated the cardiac function 22. Therefore, the effect of silibinin on oxidative stress was tested in mice underwent I/R injury. DHE staining showed a substantial generation of ROS after myocardial injury, silibinin treatment markedly decreased the fluorescence intensity of DHE (Fig. 7A, B). Moreover, compared to the vehicle, administration of silibinin significantly reduced the mRNA levels of NOX2 and NOX4 (Fig. 7C), along with higher protein expression of catalase (Fig. 7D). *In vitro* study also presented an overproduction of ROS in H9C2 cells subjected to hypoxia/reperfusion by DCFH-DA staining. In line with *in vivo* study, silibinin attenuated the fluorescence intensity of DCFH-DA after stimulation (Fig. 7E, F). Accordingly, silibinin treatment partially restored the hypoxia/reperfusion-induced dysregulation of NOX2, NOX4 and catalase in H9C2 cells (Fig. 8A, B). Meanwhile, the blunted expression of catalase was also reversed by silibinin (Fig. 8C, D).

### Silibinin inhibits I/R injury-induced inflammation

**Figure 9:**
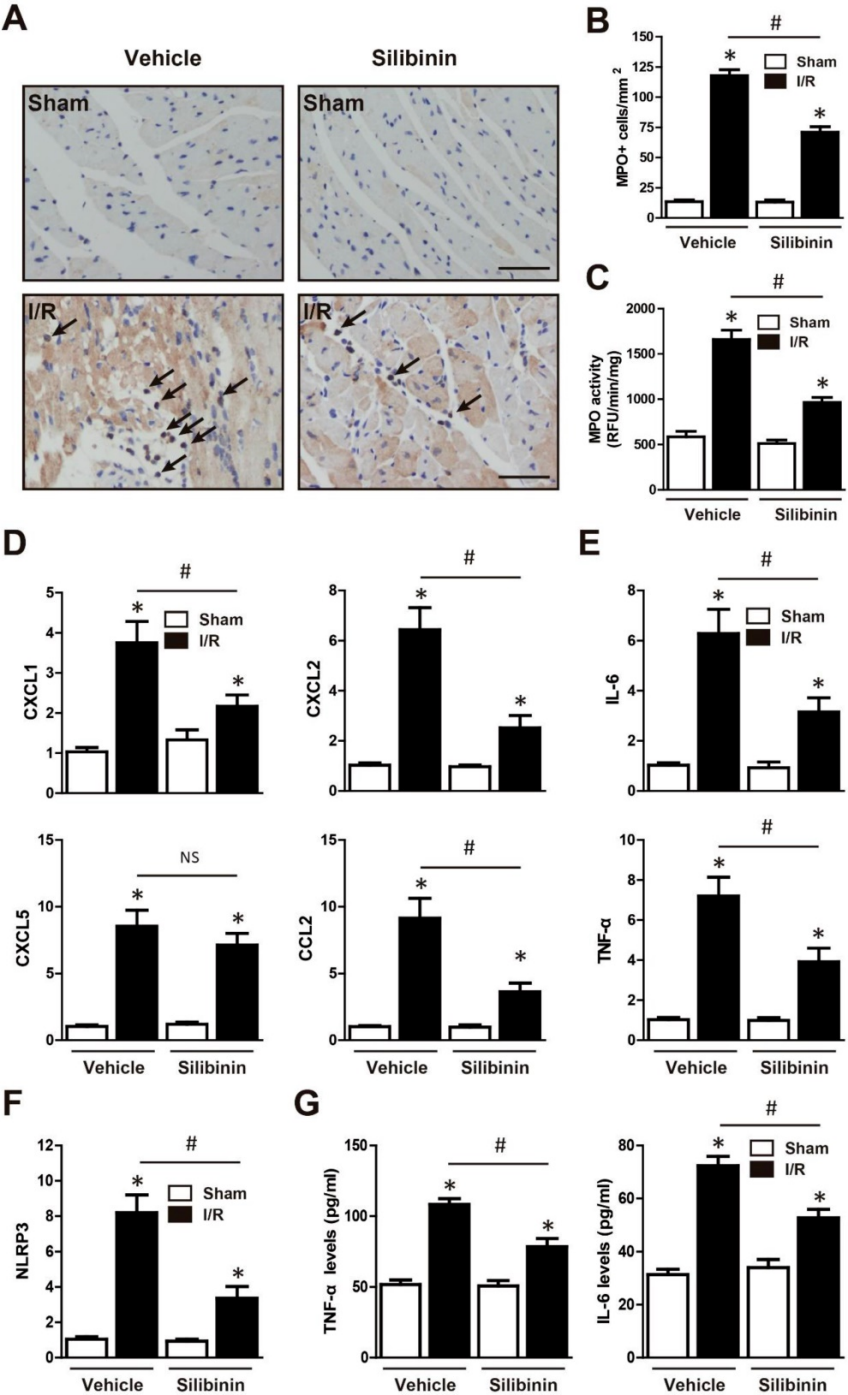
** Silibinin treatment prevents I/R-induced inflammatory response.** A, Representative immunohistochemistry images of the MPO staining in the infarct border zone (black arrow). B, Quantitative analysis of MPO-positive cells infiltrated in reperfused myocardium at 24 hours after I/R (n=6 for each). C, Measurement of MPO activity in reperfused myocardium (n=6 for each). D, CXCL1, CXCL2, CXCL5 and CCL2 transcription levels determined by real-time PCR (n=6 for each). E, IL-6 and TNF-α transcription levels determined by real-time PCR in the border zone (n=6 for each). F, Transcription level of NLRP3 determined by real-time PCR in the border zone (n=6 for each). G, the levels of IL-6 and TNF-α in the plasma after myocardial I/R injury. Bar=50 μm. *p<0.05 vs. Sham+vehicle. ^#^p<0.05 vs. I/R+vehicle. NS, not significant.

**Figure 10:**
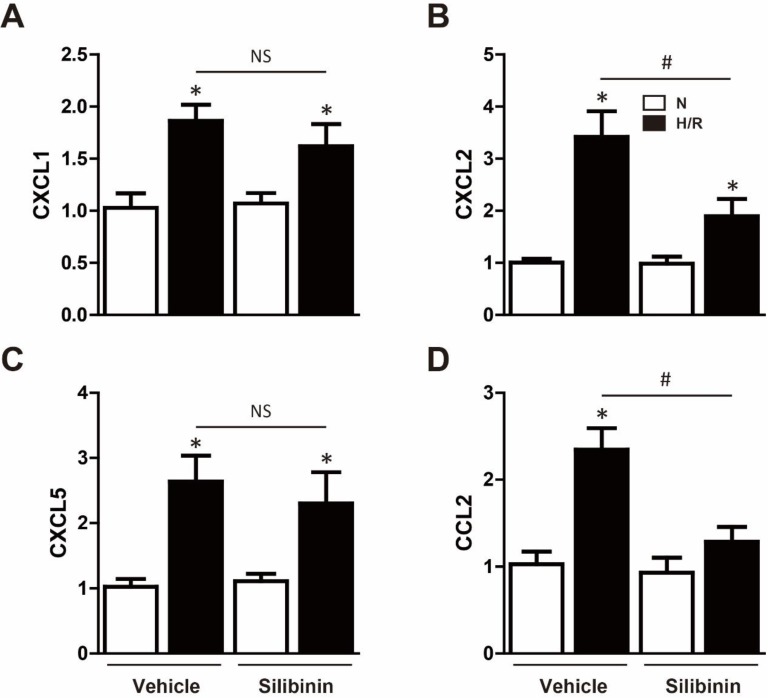
** Effect of silibinin on chemokines gene expression in H9C2 cells.** A, B, C and D, CXCL1, CXCL2, CXCL5 and CCL2 transcription levels determined by real-time PCR in H9C2 cells exposed to H/R (n=4 for each). *p<0.05 vs. Sham+vehicle. ^#^p<0.05 vs. H/R+vehicle. NS, not significant. H/R, hypoxia/reperfusion.

**Figure 11:**
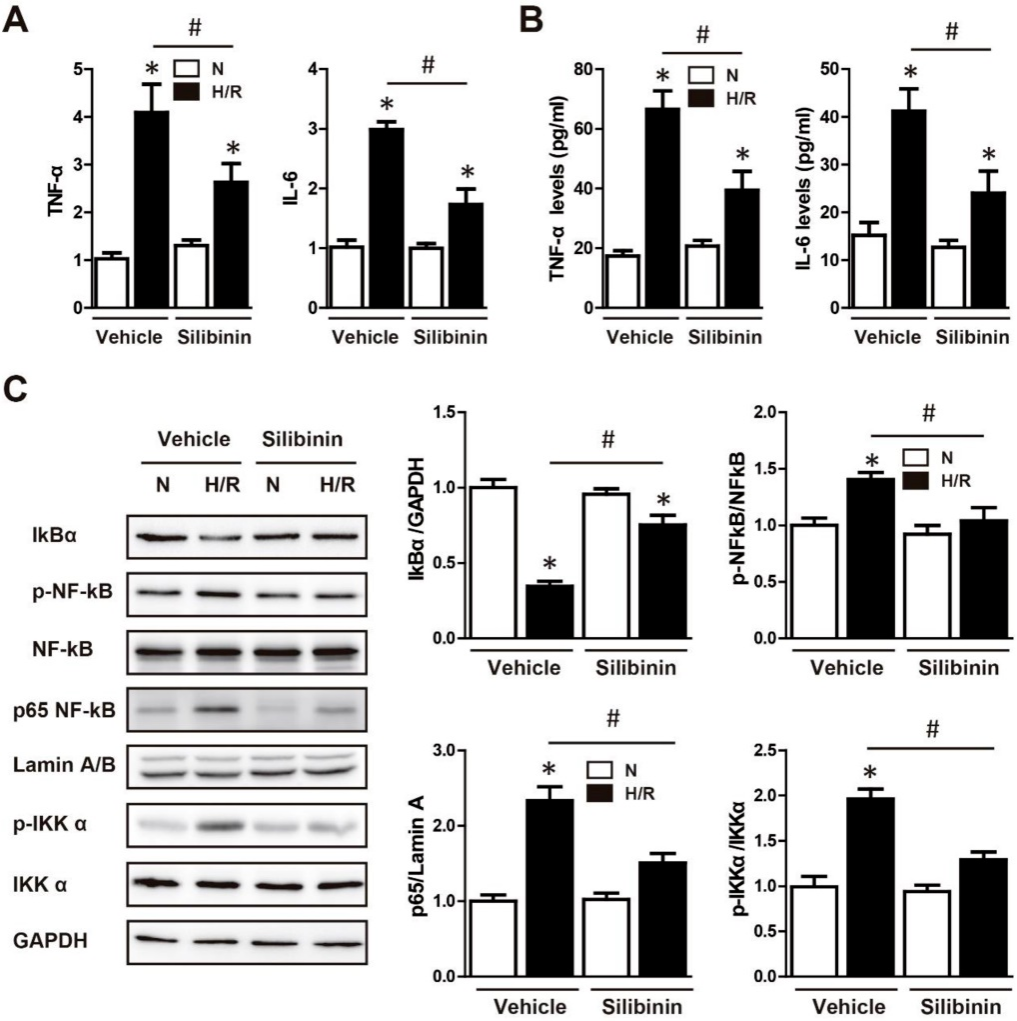
** Effect of silibinin on H/R-activated inflammatory response and NF-κB signaling in H9C2 cells.** A, IL-6 and TNF-α transcription levels determined by real-time PCR in H9C2 cells exposed to H/R (n=4 for each). B, IL-6 and TNF-α levels determined by ELISA in cultured medium after H/R (n=4 for each). C, Representative WB and quantitative analysis of IκBα, p-NF-κB, NF-κB, p65 NF-κB and p-IKKα in reperfused myocardium (n=4 for each). *p<0.05 vs. Sham+vehicle. ^#^p<0.05 vs. H/R+vehicle. H/R, hypoxia/reperfusion.

Neutrophil infiltration was not only a critical inflammatory response during I/R injury, but the major source of ROS 23. Immunohistochemistry of MPO was performed to determine the effect of silibinin in regulating inflammation. After I/R injury, the number of MPO-positive cells was significantly increased, however, silibinin treatment alleviated the infiltration of neutrophil (71±4.5% vs. 118±4.8%) (Fig. 9A, B). The activity of MPO was also diminished in mice treated with silibinin (Fig. 9C). To further investigate the role of silibinin in inhibiting neutrophil recruitment, the mRNA expression of chemokines was measured. I/R up-regulated the level of CXCL1, CXCL2, CXCL5 and CCL2, whereas silibinin treatment partially restored all of these expressions but CXCL5 (Fig. 9D). Similarly, the transcriptional levels of NLRP3 was inhibited by silibinin treatment in the context of I/R injury (Fig. 9F). Accordingly, administration of silibinin also attenuated the increase of IL-6 and TNF-α mRNA (Fig. 9E, G) from either myocardium or plasma. Furthermore, *in vitro* study confirmed the same effect in down-regulating the expression of CXCL2, CCL2, IL-6 and TNF-α in H9C2 cells exposed to hypoxia/reperfusion (Fig. 10B, D) (Fig. 11A), however with no effect on CXCL1 and CXCL5 (Fig. 10A, C). Similarly, IL-6 and TNF-α levels in cultured medium were also decreased (Fig. 11B).

### Cardioprotective effects of silibinin are dependent on deactivation of NF-κB pathway

**Figure 12:**
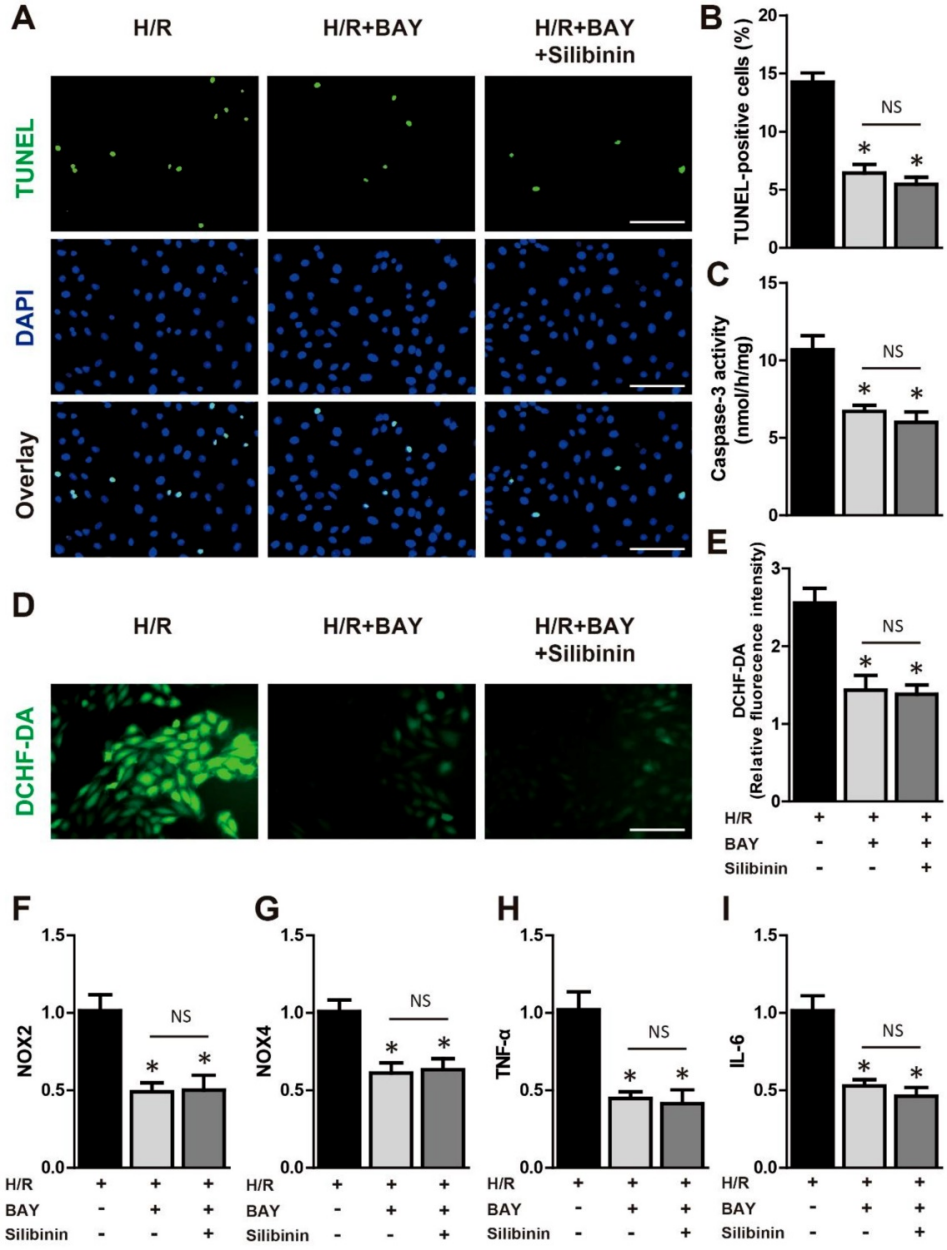
** Deactivation of NF-κB signaling mediated the protective effect of silibinin.** A, Representative immunofluorescences of TUNEL (green), and DAPI (blue) staining in H9C2 cells (n=4 for each). Bar=50 μm. B and C, Quantitative analysis of TUNEL-positive H9C2 cells and caspase-3 activity in H/R (n=4 for each). D and E, Representative images of DCHF-DA staining and quantitative analysis of fluorescence intensity in H9C2 cells (n=4 for each). Bar=50 μm. F, G, H and I, NOX2, NOX4, IL-6 and TNF-α transcription levels determined by real-time PCR in H9C2 cells exposed to H/R (n=4 for each). *p<0.05 vs. H/R+vehicle. NS, not significant. H/R, hypoxia/reperfusion. BAY, BAY 11-7082.

The NF-κB was the well-established transcript factor in regulating the inflammation, oxidative stress and cell death in the context of I/R injury 24. Western blotting showed that hypoxia/reperfusion-induced the phosphorylation of NF-κB in H9C2 cells, while silbinin treatment inhibited the expression of p-NF-κB (Fig. 12C). To further identify the effect of silibinin on deactivation of NF-κB pathway, IKKα phosphorylation, IκBα degradation and nuclear translocation of p65 NF-κB were determined. As the upstream events of NF-κB activation, the level of p-IKKα was dramatically increased, whereas the expression of IκBα was substantially decreased after hypoxia/reperfusion, treated with silibinin markedly hindered the phosphorylation of IKKα and degradation of IκBα, respectively (Fig. 12C). Similarly, nuclear protein from H9C2 cells exposed to hypoxia/reperfusion demonstrated that nuclear translocation of p65 NF-κB was completely abolished in cells treated with silibinin (Fig. 12C). To explore the critical role of NF-κB signaling involved in hypoxia/reperfusion, BAY 11-7082 (an NF-κB inhibitor) was administrated to H9C2 cells before stimulation. BAY 11-7082 treatment significantly diminished the number of TUNEL-positive H9C2 cells and activity of caspase-3 (Fig. 12A-C), suppressed the fluorescence intensity of DCFH-DA (Fig. 12D, E), and also reduced the mRNA levels of NOX2, NOX4, IL-6 and TNF-α (Fig. 12F-I). Intriguingly, silibinin plus BAY 11-7082 did not provided additive protection in attenuating myocytes apoptosis, oxidative stress and inflammatory response which demonstrated a common molecular mechanism underlying the cardioprotective effects of silibinin treatment and NF-κB signaling inhibition.

## Discussion

The present study demonstrated that silibinin treatment reduced the infarct size and serum level of cTn-I, restored the compromised cardiac function, and further retarded maladaptive fibrosis after I/R injury. Consistent with the results *in vivo*, silibinin increased the viability and ameliorated the LDH release of H9C2 cells under hypoxia/reperfusion. Subsequently, mechanistic studies identified that administration of silibinin attenuated I/R-induced cardiomyocytes apoptosis, mitochondrial dysfunction and ER stress, oxidative stress and inflammatory response. Concomitantly, treatment with silibinin hindered the activation of NF-κB pathway while additional BAY 11-7082 did not provide further protection in culture H9C2 cells under hypoxia/reperfusion, which revealed a potential molecular target for the cardioprotective effect of silibinin. Taken together, our study first provided direct evidence that silibinin exerted powerful protection against myocardial I/R damage.

I/R injury was currently the crucial determinant of cardiomyocyte death in the setting of restored coronary flow by PCI or thrombolytic therapy in patients suffered from acute MI 1. Numerous studies revealed that apoptosis, oxidative stress, intracellular Ca2+ overload, mPTP opening, along with inflammation were deeply involved in the pathological progression and thus provided the rationale for therapeutic strategies targeted against these adverse pathways 2-4,25. It had been demonstrated that apoptosis, which triggered via the intrinsic and extrinsic pathways, directly mediated the deterioration of cardiac function 26. The former was mainly triggered by cytochrome c released from impaired mitochondria, followed by cleaved procaspase-9 and caspase-3 activation. In our study, silibinin significantly inhibited the intrinsic apoptotic process post myocardial I/R injury, evidenced by less release of cytochrome c and reduced activity of caspase-9/3. Concomitantly, we found that silibinin treatment also mitigated the I/R-induced ER stress and corresponding pro-apoptotic signaling pathway. Misfolded protein secondary to cardiac disease caused ER stress, increased the expression of CHOP and promoted caspase-12 dependent apoptosis 17,27,28. Simultaneously, ER stress disrupted the redox balance, resulted in ROS accumulation and mitochondria dysfunction, finally accentuated cardiomyocytes apoptosis 29,30. However, the detailed mechanisms underlying the effects of silibinin against the viscous cycle among ER stress, mitochondria impairment and apoptosis remained unclear. In addition, impaired mitochondria and ER stress led to a burst of ROS, which in turn exacerbated the dysfunction of itself and induced cardiomyocytes apoptosis 26,29. Silibinin was the well-established antihepatotoxic agent in clinical practice, which predominantly prevented from oxidative stress by scavenging free radicals 10,16. Expectedly, administration of silibinin reduced the level of ROS both *in vivo* and *in vitro*. Meanwhile, as an important enzyme in regulation of H_2_O_2_ or O_2_-, NADPH oxidases maintained the homeostasis of cell death 31,32. Prior experiments had reported that genetic deletion or pharmacological inhibition of NOX2 or NOX4 reduced ROS production and infarct size in the context of cardiac disease 33,34. Conversely, cardiac-specific overexpression of NOX2 elevated the ROS level, increased cross-sectional area of cardiomyocytes and collagen deposition, and eventually aggregated LV dysfunction post MI 35. As shown in present study, the transcript levels of NOX2 and NOX4 were markedly increased in reperfused myocardium and partially alleviated by silibinin treatment. Taken together, these findings also indicated the powerful antioxidant effects of silibinin in reperfusion injury.

Vigorous inflammation subsequent to oxidative stress played an important role in myocardial I/R injury 4,36,37. Substantial chemokines, cytokines generated by cardiomyocytes recruit inflammatory cells to reperfused myocardium in the early phase 2. Neutrophils infiltration was the hallmark of inflammatory response exposed to pathological condition and facilitated I/R injury by releasing proteolytic enzymes 23. It was noteworthy that activated neutrophils were a critical source of ROS and therefore augmented oxidative stress 38. In line with this, our study observed a dramatic increase of neutrophils within injured myocardium, whereas silibinin treatment alleviated the infiltration of neutrophils. Moreover, CXC chemokines were a prerequisite for neutrophils migration and activation 39. *In vivo* experiments also showed an inhibited generation of CXCL1, CXCL2 and CCL2 with silibinin treatment, and a similar tendency was identified *in vitro* hypoxia/reperfusion model. Meanwhile, silibinin concomitantly reduced the expression of pro-inflammatory cytokines. Notably, pro-inflammatory cytokines were extensively studied in diverse cardiovascular diseases, pharmacologic or genetic intervention could attenuate adverse ventricular remodeling and cardiac dysfunction 40-43. Thus, silibinin exerted cardioprotective effects in myocardial I/R damage were partially via suppression of inflammatory signaling cascades.

It was well accepted that NF-κB transcription factor played a pivotal role in modulation of inflammation, oxidative stress and cell death in cardiac pathology 24,44. Activation of NF-κB signaling pathway depended on proteasomal degradation of IκB and subsequent nuclear translocation of NF-κB, where phosphorylated NF-κB regulated the expression of target gene 19. As reported in a previous study, blocking NF-κB signaling by specific inhibitor limited the infarct size, reduced the troponin-I release and preserved cardiac function in reperfused myocardium 24. Furthermore, genetic inhibition of NF-κB by cardiac specified expression of mutant IκBα also presented smaller infarct size despite no difference in echocardiographic parameters after I/R injury 45. Consistently, our study demonstrated that silibinin restrained IκBα degradation and p65 NF-κB translocation into nucleus, which contributed to the deactivation of NF-kB signaling pathway. Therefore, this work implied that inhibition of NF-κB activation cascades mediated the protective effects of silibinin in the setting of reperfusion insult.

## Conclusion

Silibinin was a novel cardioprotective agent against myocardial I/R injury by inhibiting cardiomyocytes apoptosis, reducing ER stress and oxidative stress, and modulating inflammatory response via deactivation of NF-kB signaling pathway. Present study revealed the molecular mechanisms responsible for the beneficial effects of silibinin and provided a promising therapeutic approach for the treatment of myocardial I/R injury.

## Figures and Tables

**Table 1:** The information of primers.

Gene	Species	Forward (5'→3')	Reverse (5'→3')
BNP	Mouse	GAGGTCACTCCTATCCTCT	GCCATTTCCTCCGACTTTTCTC
NOX2	Mouse	TGTGGTTGGGGCTGAATGTC	CTGAGAAAGGAGAGCAGATTTCG
NOX4	Mouse	GAAGGGGTTAAACACCTCTGC	ATGCTCTGCTTAAACACAATCCT
CTGF	Mouse	GGGCCTCTTCTGCGATTTC	ATCCAGGCAAGTGCATTGGTA
PAI-1	Mouse	TTCAGCCCTTGCTTGCCTC	ACACTTTTACTCCGAAGTCGGT
CXCL1	Mouse	CTGGGATTCACCTCAAGAACATC	CAGGGTCAAGGCAAGCCTC
CXCL2	Mouse	CCAACCACCAGGCTACAGG	GCGTCACACTCAAGCTCTG
CXCL5	Mouse	TCCAGCTCGCCATTCATGC	TTGCGGCTATGACTGAGGAAG
CCL2	Mouse	TTAAAAACCTGGATCGGAACCAA	GCATTAGCTTCAGATTTACGGGT
IL-6	Mouse	TAGTCCTTCCTACCCCAATTTCC	TTGGTCCTTAGCCACTCCTTC
TNF-α	Mouse	CCCTCACACTCAGATCATCTTCT	GCTACGACGTGGGCTACAG
NLRP3	Mouse	ATTACCCGCCCGAGAAAGG	TCGCAGCAAAGATCCACACAG
CXCL1	Rat	CCGCTCGCTTCTCTGTGCAG	ACCATTCTTGAGTGTGGCTATGACTTC
CXCL2	Rat	ATGCTGTACTGGTCCTGCTCCTC	GTCACCGTCAAGCTCTGGATGTTC
CXCL5	Rat	CAGAGAGGTGGTGGTGGTGA	CCCGTTCTTCAGGGAGGCTA
CCL2	Rat	TAGCATCCACGTGCTGTCTC	TGCTGCTGGTGATTCTCTTG
IL-6	Rat	AAGCCAGAGTCATTCAGAGCAATACTG	GATGAGTTGGATGGTCTTGGTCCTTAG
TNF-α	Rat	TCCAGAACTCCAGGCGGTGTC	GTTCAGTAGACAGAAGAGCGTGGTG
NOX2	Rat	ACTTCTTGGGTCAGCACTGG	GTTCCTGTCCAGTTGTCTTCG
NOX4	Rat	TAGCTGCCCACTTGGTGAACG	TGTAACCATGAGGAACAATACCACC

## Abbreviations

I/R: ischemia/reperfusion
LAD: left anterior descending coronary artery
AAR: area at risk
LV: left ventricle
LVESD: left ventricular end-systolic dimension
LVEDD: left ventricular end-diastolic dimension
LVEF: left ventricular ejection fraction
FS: fraction shorting
ROS: reactive oxygen species
cTn-I: cardiac troponin I
ER: endoplasmic reticulum
ELISA: enzyme linked immunosorbent assay
LDH: lactate dehydrogenase
CHOP: CCAAT/enhancer-binding protein homologous protein
mPTP: mitochondrial permeability transition pore
NF-κB: nuclear factor κB
BNP: brain natriuretic peptide
TTC: triphenyltetrazolium chloride
TUNEL: TdT-mediated dUTP Nick-End Labeling
OCT: optical coherence tomography
MPO: myeloperoxidase
PAI-1: plasminogen activator inhibitor-
CTGF: connective tissue growth factor
